# Screening for Predictors of Chronic Ciguatera Poisoning: An Exploratory Analysis among Hospitalized Cases from French Polynesia

**DOI:** 10.3390/toxins13090646

**Published:** 2021-09-12

**Authors:** Clémence Mahana iti Gatti, Kiyojiken Chung, Erwan Oehler, T. J. Pierce, Matthew O. Gribble, Mireille Chinain

**Affiliations:** 1 Laboratory of Marine Biotoxins, Institut Louis Malardé (ILM), UMR 241-EIO (IFREMER, ILM, IRD, Univ. Polynésie Française), Papeete, Tahiti 98713, French Polynesia; kiyo.chung@outlook.com (K.C.); mchinain@ilm.pf (M.C.); 2 Centre Hospitalier de Polynésie française, Papeete, Tahiti 98713, French Polynesia; erwan.oehler@cht.pf; 3 Gangarosa Department of Environmental Health, Department of Epidemiology, Emory University Rollins School of Public Health, Atlanta, GA 30322, USA; tjpierce223@gmail.com; 4 Department of Epidemiology, University of Alabama at Birmingham, Birmingham, AL 35233, USA; mgribble@uab.edu

**Keywords:** ciguatera poisoning, epidemiology, least absolute shrinkage and selection operator, machine learning, data science, medical informatics, survival analysis, foodborne diseases

## Abstract

Ciguatera poisoning is a globally occurring seafood disease caused by the ingestion of marine products contaminated with dinoflagellate produced neurotoxins. Persistent forms of ciguatera, which prove to be highly debilitating, are poorly studied and represent a significant medical issue. The present study aims to better understand chronic ciguatera manifestations and identify potential predictive factors for their duration. Medical files of 49 patients were analyzed, and the post-hospitalization evolution of the disease assessed through a follow-up questionnaire. A rigorous logistic lasso regression model was applied to select significant predictors from a list of 37 patient characteristics potentially predictive of having chronic symptoms. Missing data were handled by complete case analysis, and a survival analysis was implemented. All models used standardized variables, and multiple comparisons in the survival analyses were handled by Bonferroni correction. Among all studied variables, five significant predictors of having symptoms lasting ≥3 months were identified: age, tobacco consumption, acute bradycardia, laboratory measures of urea, and neutrophils. This exploratory, hypothesis-generating study contributes to the development of ciguatera epidemiology by narrowing the list from 37 possible predictors to a list of five predictors that seem worth further investigation as candidate risk factors in more targeted studies of ciguatera symptom duration.

## 1. Introduction

Ciguatera poisoning (CP) is a non-infectious food poisoning resulting from the consumption of marine organisms contaminated by ciguatoxins (CTXs), neurotoxins produced by microalgae in the genera *Gambierdiscus* and *Fukuyoa*, that preferentially proliferate in tropical and subtropical ecosystems [1,2]. According to current figures, this would affect 10,000 to 50,000 persons every year [3], nevertheless, it is likely that these numbers only represent 20% of the true situation due to the absence of diagnostic tools, and a strong under reporting of the disease at a global scale [4]. Due to the nature and the large distribution of CTXs’ biological targets (e.g., voltage-gated sodium channels, potassium channels, and calcium channels) and the variety of cells affected [5,6,7,8], CP results in a variable and highly subjective syndrome. Indeed, symptom expression and severity may vary [9] under the combined influence of the amount, and likely, the family of CTXs ingested, as well as individual susceptibility. CP diagnosis is solely based on the history of poisoning and symptoms’ description, and must be considered in a non-febrile patient presenting with a combination of gastrointestinal, cardiovascular, musculoskeletal and/or neurological disorders (especially cold allodynia, unsustainable itching and heightened nociperception), which appear within 48 h after the ingestion of “at-risk” marine organisms [9]. Gastrointestinal manifestations (i.e., diarrhea, vomiting, nausea, and abdominal pain) usually occur first, sometimes accompanied by cardiovascular disorders, mostly bradycardia and hypotension. These manifestations generally disappear within 48–72 h, either spontaneously or under adapted medication, while neurological and other systemic manifestations (e.g., cold allodynia, itching, paraesthesia, myalgia, arthralgia) appear during subsequent days [10,11,12,13].

Chronic forms of CP with symptoms lasting ≥3 months are a real clinical challenge. Even though chronic CP cases are regularly reported, studies dedicated to the understanding of this phenomenon are scarce, especially due to the lack of a clear consensus regarding its clinical definition. Indeed, some CP manifestations may persist for months or even years after the initial poisoning, mostly under the traits of neurological and psychiatric disorders (i.e., tenacious fatigue, paraesthesia, dysesthesia, pruritus, attention deficit disorder, anxiety, depression) [14,15,16,17]. These manifestations can be expressed continuously or through transient reactivation peaks, triggered by multiple factors (e.g., consumption of marine/fresh water-related products, even from non-endemic region, alcohol, nuts, and red meat. Stress and contrasted ambient temperature may also impact [9,17]. Diagnosing a chronic CP, with a time lag from the initial poisoning, represents a real struggle for both patients and clinicians, as it often required costly and unsatisfactory biological tests. To date, no treatment has proven effective in CP medical management. Treatment essentially relies on supportive care, with variable results [9,18]. Considering that at least 20% of the persons affected by CP are likely to develop a persistent form [19], this health issue deserves an increased attention and dedicated studies.

The present study aims at better characterizing CP chronic forms among a cohort of individuals that have been hospitalized for CP motive at the main hospital of French Polynesia (FP), a territory with a high CP endemicity, and assessing a statistical predictive model for symptoms’ duration.

## 2. Results

### 2.1. Participants’ Description

Among the n = 49 participants, n = 26 were identified as acute CP cases (with symptoms that fully improved in less than 3 months) and n = 23 as chronic CP cases. As shown in Table 1, the median age was 50 years (47 years old in acute cases vs. 52 years old in chronic cases); and males were predominant (SR_n=49_: 1.882; SR_acute_: 1.364; SR_chonic_: 2.833). The cohort was composed of 65% (n = 32) Polynesian, 26% (n = 13) mixed-race and 8% (n = 4) non-Polynesians. A total of 52% (n = 12) of chronic patients had one or more comorbidities such as chronic hypertension and type 2 diabetes vs. 11% (n = 3) among the acute CP cases; and 65% (n = 15) of chronic CP cases declared weekly tobacco consumption at the time of the poisoning vs. 23% (n = 6) among acute CP cases.

### 2.2. Acute Symptoms and Biological Variables

In Table 2 we reported the acute symptoms and biological characteristics of the n = 49 participants (or the subset of participants with complete data on that variable) vs. the n = 38 participants included in the rigorous logistic lasso regression (RLLR model). For binary variables, the percent reporting “yes” is reported; for continuous variables, the median (25th percentile, 75th percentile) is reported. The symptoms incubation time was once recorded as “>3 h”, and once as “<24 h”; for data analysis, these were assumed to fall into the categories of <12 h and ≥12 h, respectively.

### 2.3. Chronic Symptoms and Triggering Factors

As shown in Table 3, the most prevalent chronic symptoms were neurological (i.e., itching (65%), asthenia (61%), paraesthesia (56%), cold allodynia (39%), dysesthesia (26%), muscular disorders (26%) and blurred vision (26%)), whereas digestive and cardiovascular manifestations were less present (diarrhea (17%) and peak of hypotension (13%)). As to neuropsychiatric manifestations such as sleep disorder, irritability, depression, and attention disorders, they were expressed with a respective prevalence of 22%, 17%, 9%, and 4%.

Those manifestations were reported to be triggered or worsened by factors that mainly belong to food products category, such as fish (56%), chicken (30%), beef (26%), nuts (22%), pork (17%), soy related products (17%), canned food (17%), dairy products (17%), alcohol (13%), coffee/tea (9%), chocolate (9%) and spices (4%) (Table 4). Note that the prevalence for fish, meat, and alcohol may be lowered by the fact that some of the participants avoided them following their hospital discharge, under the recommendation of their physician, and still did not reintroduce them in their diet when the interview was realized. Non-food factors were also reported to influence chronic symptoms’ expression, such as cold/hot ambient air contrast (n = 4), rapid weight loss (n = 4), physical activity (n = 3), and sun exposure (n = 4).

### 2.4. Chronic CP Predictors

Table 5 provides the *p* value from either a Fisher’s exact test for categorical predictors (e.g., tobacco consumption, acute bradycardia symptoms) or a *t*-test for continuous predictors (e.g., age, urea, neutrophils) comparing cases to non-cases of chronic CP. There were n = 49 included in tests of the binary predictors, and n = 45 participants with complete data on laboratory biomarkers for two sample equal-variance *t*-tests. Only the comparison of tobacco consumption between the cases and non-cases of chronic CP was Bonferroni-significant (*p* < 0.01) considering the 5 hypothesis tests. Unlike the RLLR analysis, these hypothesis tests did not use standardized variables.

A two-by-two table of the observed chronic case status vs. the predicted chronic case status given the RLLR model, with model predictions calculated for all n = 49 participants is presented in Table 6. There were n = 35 patients for whom the model prediction matched the observed chronic case status vs. n = 14 for whom the model prediction did not match. The sensitivity of this prediction model was 92% (95% confidence interval: 75%, 99%) and the specificity of this prediction model was 48% (95% confidence interval: 27%, 69%). The positive predictive value of this model was 67% (95% confidence interval: 57%, 75%), and the negative predictive value of this model was 85% (95% confidence interval: 58%, 96%). The model accuracy was 71% (57%, 83%).

### 2.5. Time until Cessation of Symptoms among Chronic Cases

The smallest sample-size survival model had 20 participants, 13 with observed cessation of symptoms during follow-up, and 450 total months of follow-up.

The results of the parametric survival analyses of time until cessation of symptoms among chronic cases, considering only the variables suggested as predictive of being a chronic case in the RLLR model, are presented in Table 7. There were 16 chronic cases with recorded cessation of symptoms and 7 who had not yet experienced a cessation of symptoms at the end of the study follow-up period (total n = 23). The maximum analysis time at risk in this survival analysis was 535 person-months (n = 23 participants, n = 16 events) and the minimum was 450 person-months (n = 20 participants, n = 13 events). Note that standardized variables were used for this predictive analysis. One of the bootstrap estimates for the lognormal survival-time model with bradycardia as the predictor failed to converge so the confidence interval was based on 499 bootstrap replicates.

## 3. Discussion

Among the n = 49 participants, 47% (n = 23) kept suffering from persisting symptoms at least 3 months after the initial poisoning. However, this number may be biased as it can be hypothesized that candidates who developed chronic manifestations were more inclined to contribute to the study. As comparison, Pearn [19] and Baumann et al. [20] have respectively estimated this prevalence at 20% and 34%.

The acute clinical picture of our cohort is comparable with the one described by Gatti et al. [21]. Indeed, among the n = 129 hospitalizations for ciguatera registered at the same hospital between 1999 and 2005, the most prevalent symptoms were identical, i.e., diarrhea, vomiting and abdominal pain regarding gastrointestinal symptoms; hypotension and bradycardia, as cardiovascular symptoms; and paraesthesia, asthenia and pruritus among neurological symptoms.

Moreover, 43% of the participants in the present study declared smoking tobacco (vs. 41% of the population of French Polynesia in 2010 [22]), suffering from chronic hypertension for 16% of them (vs. 26.7% [22]) and type 2 diabetes for 16% of them (vs. 19% in 2012 [23]). In addition, the prevalence of participants with antecedent of ciguatera (57%) is in accordance with the estimations observed among ambulatory cases recorded through the surveillance program of FP (51.6% in 2016 [24], 56% in 2020 [25]). This information suggests that the study cohort does not present higher risk or fragility than the general population.

### 3.1. Chronic Manifestations and Triggering Factors

Chronic manifestations, that are mainly dominated by neurological symptoms, are also consistent with observations made in a 20 months’ follow-up of 9 tourists contaminated with trochus shell, *Tectus niloticus* (whose specimens from the same site were showed to contain high amounts of CTXs) [17,26]. Indeed, itching, asthenia, paranesthesia and cold allodynia were also the most prevalent persisting symptoms which gives CP its status as mixed sensory neuropathy that predominantly affects the lemniscal system [12,27], while cardiovascular and digestive manifestations were absent or anecdotal. Note that in both studies, neuropsychiatric manifestations essentially made their appearance during the chronic stage.

There are several mechanisms that were given as explanations for the persistence of CP symptoms. It has been hypothesized that it could result from prolonged activation of voltage-gated sodium channels by CTXs, fixed in deep tissues and occasionally released into the bloodstream following lipid metabolism activation [28], and/or a possible immune imbalance common to several chronic auto-inflammatory and auto-immune diseases [29,30,31,32,33]. In the present study, the first theory is supported by the declaration of symptoms worsening after non-rapid weight loss in 4 participants. Concerning neuropsychiatric manifestations such as depression, anxiety, memory loss or attention disorder [11,34,35,36], they could be attributable to (i) a pre-existing non-CP related psychiatric illness, (ii) a secondary consequence of a certain lassitude linked to persistent invalidating manifestations that could not be mitigated or removed by effective treatments, and a lack of recognition by medical professionals, (iii) to direct physiological damages caused by CTXs on the central nervous system, or (iv) to a synergistic effect of CTXs biological impacts combined with functional/psychiatric disorders. As also observed in the Gatti et al. 20 month’s follow-up study [17], except for psychiatric-related symptoms that can make their appearance weeks or months after the initial poisoning, no new symptoms other than the ones expressed during the acute phase made their appearance.

As also observed in the present study, characteristic of CP is that its symptoms, especially neurological ones, may be modulated by external factors contributing to transient relapse or intensification. [9]. Although many symptom-triggering factors reported by the ciguatera cases in this study (Table 4) have a food-based origin, others are related to the environment (e.g., contrasted ambient air temperature, sun exposure) or linked to patients’ physical condition (e.g., rapid weight loss, intense physical activity). Assembling a list of potentially modifiable triggering factors would be helpful to assist CP patients in their recovery process by adapting their diet and behavior. It is possible that mechanism for symptom triggering might be related to histamine metabolism, due to the similarities that can be observed in CP patients and certain histamine-related disorders, although this has not been assessed in this study.

Finally, in this study as also observed in Chateau-Degat et al. [16] and Gatti et al. [17], CP usually showed a positive evolution, with symptoms that progressively and spontaneously regressed in frequency and intensity over time.

### 3.2. Chronic CP Predictive Factors

In this exploratory study, our RLLR model identified five potentially predictive variables of developing a chronic ciguatera form that would merit further investigation: age, tobacco consumption, acute bradycardia, laboratory measures of urea and neutrophils. This predictor-screening RLLR model used a standardized version of each predictor variable rather than the real-world untransformed observed values of each variable. When we considered untransformed variables, only tobacco consumption was Bonferroni-significant when considered in relation to observed chronic case status. In secondary survival analyses for duration of symptoms among chronic cases, the point estimates for tobacco consumption were consistent with an extended duration of symptoms among tobacco consumers, but these model estimates were imprecise and not statistically significant. Nevertheless, this finding is consistent with the observation of Chateau-Degat et al. [16] who identified a clear association between tobacco consumption and CP symptoms persistence on mild CP cases. Further toxicological research on tobacco implication (through a co-modulation of cholinergic receptors by CTXs and nicotine), as a potential obstacle in the healing process, is warranted.

Acute hypotension and bradycardia were respectively expressed in 80% and 55% of participants, which reinforces the idea that our study population suffers from a severe form of CP. Indeed, according to Geller and Benowitz [37], these manifestations would result from an autonomic dysfunction (combination of parasympathetic excess and sympathetic failure), considered one of the most serious CP complications, also used as determining factor for patient’s hospitalization. By comparison, hypotension and bradycardia prevalence among CP ambulatory cases reported in French Polynesia were respectively 19.7% and 24.3% in 2018 [38]. Bradycardia as predictive factors for chronic cases could mean that the severity of the poisoning would have an influence on patients’ recovery capacity. At this stage, a prospective comparative study between ambulatory and hospitalized cases, with the support of a severity index, may be able to answer this question.

Our study also suggested that age may be a predictive factor. Such observation is consistent with the result of Katz et al. [39] who found a correlation between age and increasing duration of CP symptoms while describing an outbreak in Hawaii. This finding has biologically plausible mechanisms relating to the decreasing capacity of neuronal repair and/or a slower sodium channel regeneration with age, accentuated by the ability of some CTX to reduce the intrinsic growth capacity of peripheral neurons as observed on animal model [40].

Known to be a significant actor of the innate immune response, neutrophils are also implicated in several other processes, such as acute cell injury and repair, cancer, autoimmunity or chronic inflammation [41]. Even though the innate immune response to non-protein toxins is poorly documented, there is some evidence that CTXs are involved in both immune and inflammatory dysregulation [7,31]. As mentioned by Pierre et al. [42], available data suggest that the systemic immune response following acute CTX exposure is rather an anti-inflammatory Th2 response that may result in a possible genetic predisposition associated with MHC variants conferring susceptibility to develop a persistent syndrome. As these processes are not clearly established yet, we are unsure about the mechanism by which neutrophil regulation is related to CP symptoms duration. These findings merit further dedicated studies.

Urea, a nitrogenous metabolite resulting from the catabolism of proteins, is mainly measured to assess kidney function, even though its production may be easily influenced by several unconnected medical causes [43]. At this stage, suggesting a significant biological explanation for its role as a potential predictive factor for symptoms duration remains nebulous and needs further research.

It is possible that some of these factors, although they did not show detectable associations in this sample, could have a causal relationship that might be detectable in a larger sample; for example, it is possible that diabetes may contribute to more sensitive nerve cells to the variations of osmolarity induced by CTXs, and consequently to more substantial neurological disturbances.

### 3.3. Limitations of the Study

As in any study based on the recall of past events, recall bias represents a significant weakness, which, however, was partially controlled by comparing data collected through questionnaires with data from hospital medical records. Yet, and above all, the main weakness resides in the small sample size of the cohort, especially given the population initially targeted (n = 254 potential candidates). Another limitation of this study resides in the fact that it was focused on screening individual predictors and did not allow for possible interactions between predictors. This screen suggested that tobacco consumption may be of particular interest for follow-up research as a potentially modifiable, significant predictor of being a chronic case; we do not evaluate in this study possible heterogeneity in the association of tobacco with ciguatera duration according to sex or any other participant characteristics.

Overall, there was a good agreement of our RLLR model prediction of the most likely outcome per participant vis-à-vis the observed outcomes. The overall model accuracy was 71%, which can likely be improved if additional predictive variables are identified in the future, but suggests that these five variables may be informative for risk stratification of new ciguatera patients as having elevated risk of becoming a chronic case. The negative predictive value of this model was better than the positive predictive value, and the sensitivity was better than the specificity.

The survival analysis examining the associations of these five variables to the duration of symptoms is conditional on the results of the original RLLR model with a somewhat related outcome variable, as well as having five separate hypothesis tests, and so would be expected to also have an inflated Type I error rate. We find a nominally significant (*p* < 0.05), but not Bonferroni-significant, association of higher levels of (standardized) urea with shorter duration of symptoms, assuming a Weibull distribution for survival times. However, this is no longer nominally significant when we consider a lognormal distributional assumption for the survival-time distribution. Thus, our inferences about statistical significance or lack thereof are sensitive to our parametric model assumptions, although we obtained similar point estimates for each predictor variable regardless of distributional assumptions. Our subjective impression is that both urea and bradycardia merit further investigation as potential predictors of the timing of cessation of symptoms in a future study. We intentionally limited our number of hypothesis tests due to our very limited sample size for the survival analyses. Since we used RLLR for chronic ciguatera as a screening tool to reduce the number of modeled covariates, we cannot preclude the possibility that another variable that was not identified in the RLLR model as significantly predictive would nevertheless be predictive of the duration of symptoms conditional on being a chronic case.

Despite the lack of knowledge about the mechanisms underlying CP, it appears important to bring to the attention of healthcare workers the existence of CP chronic manifestations, especially in a context of globalization of this affliction [44]. Moreover, collaborative international efforts must be encouraged in order to (i) increase CP awareness, especially in non-endemic regions, (ii) define a “Medical Consensus Guideline” to come in support of the medical community, (iii) develop diagnosis tools and effective treatments. Although the conclusions of the present study are very preliminary, based on a limited sample size and mixed findings, it may be of valuable interest in order to narrow the search space for future investigations from an agnostic set of 37 potential predictors to a more feasible study set of 5 candidate predictors with suggestive but not conclusive prior evidence for association.

## 4. Materials and Methods

### 4.1. Cohort Recruitment and Data Collection

Candidates to the study were selected from the medical records register of patients hospitalized for ciguatera motive (coded T61.0 according to the International Classification of Diseases), between 1 January 2010 and 31 December 2015, at the Centre Hospitalier de Polynésie Française. All T61.0-corresponding files were then verified by the CP referent physician of the hospital for a posteriori diagnosis confirmation. After excluding (i) incorrectly coded cases, (ii) persons that have left the territory, (iii) deceased persons, (iv) people with serious physical or mental condition incompatible with the holding of an interview, (v) and people who could not be contacted due to false or no contact information; n = 100 potential candidates were identified to be included in the study and met the investigators for a follow-up interview. Among them, n = 13 refused to participate and n = 38 never showed up to the follow-up interview despite giving their consent. The study was finally conducted on 49 participants for whom we disposed of acute ciguatera hospitalization medical records (which dates back to 3 months at the earliest) and chronic symptom’s evolution collected through a dedicated questionnaire carried out in face-to-face or remotely with the assistance of the investigators when needed. In detail, medical records gathered information about patients’ characteristics (age, sex, medical history, tobacco consumption, comorbidities), the context of poisoning, the acute clinical description and biological test results (carried out systematically for all incoming patients regardless of the reason for their hospitalization). The follow-up questionnaire included questions related to the participant medical history, the initial poisoning context, the acute symptoms during the hospitalization (in order to assess the level of reliability of their memories, by comparing this information with that contained in the hospital medical records), the chronic evolution of symptoms, and the factors likely to influence their expression.

### 4.2. Statistical Analysis and Predictive Model

Lasso regression [45] is a well-established machine learning approach that uses regularization to stabilize model estimation by solving for optimal regression coefficients $\hat{\beta }$ to minimize a loss function $-\frac{2}{n}\mathrm{LL}\left(\beta \right)+\lambda \underset{j}{\sum }\left|\beta_{j}\right|$, where LL represents the log-likelihood of the model for coefficient set β, n is the sample size, λ is a model tuning parameter that determines how much penalty to attach to extra model complexity (i.e., inclusion of nonzero coefficients), and $\beta_{j}$ is each regression parameter. The lasso regression approach lacks a formal causal interpretation but can be useful for discerning predictive variables in situations where there is data sparsity (e.g., a large number of candidate predictors compared to the number of observations, with the number of predictors potentially exceeding the number of observations). The primary goal of lasso regression is prediction rather than causal inference; it is possible that a variable that is retained in the model as predictive is correlated with a causal variable but is not a causal variable.

Rigorous logistic lasso regression [46], is an extension of the lasso modeling framework developed to handle binary outcome variables with potentially heteroskedastic errors or clustering of observations and a limited sample size relative to the number of observations. The penalty in RLLR is defined by the equation $\lambda =\frac{c}{2}\sqrt{n}\Phi^{-1}(1-\gamma$), where c is a slack parameter (we constrained to c = 0.1), Φ is the Gaussian cumulative distribution function, and γ is the significance level (we constrained to $\gamma =\frac{0.05}{\max\left(p*\log\left(n\right),n\right)}$). Thus, using this constrained approach there was only a single λ possible and no grid search was needed for an optimal λ.

For the RLLR model, we included biological test that had been realized in ≥90% of participants (for urea, creatinine, sodium, potassium, chloride, leukocytes, neutrophils, eosinophils, basophils and lymphocytes measures); whether the patient had consumed herbivorous fish or a different kind of seafood; and whether or not the patient had consumed a part of the seafood more likely to be high in CTXs (i.e., head or viscera). We excluded participants missing data on any of these variables, leading to a reduced sample size of n = 38, and considered acute symptoms that were observed in >10% (n ≥ 4) and <90% (n ≤ 34) of the n = 38 patients with complete data on the included biological tests and fish-related variables. We also considered participant age, sex, ciguatera incubation time (dichotomized at ≥12 h, with the assumption that a recorded “>3 h” meant <12 h, and a recorded “<24 h” meant ≥12 h), previous ciguatera, chronic hypertension, diabetes status, and tobacco consumption.

Thus, there were a total of n = 38, p = 44 included in the RLLR. All these candidate predictors were standardized prior to the RLLR to allow for comparable-magnitude regression coefficients. The RLLR models were implemented in Stata version 16.1 M/P software using the <lassopack> contributed by Ahrens, Hansen, and Schaffer [47]. We subsequently evaluated the predictive ability of the RLLR model by calculating the most likely outcome (i.e., chronic ciguatera case or not) model predictions in Stata for all n = 49 participants. All predictors were included in one model.

We then considered whether the significant candidate predictors of being a CP chronic case in the RLLR model were individually associated with being a chronic case in the larger patient sample with complete data on the candidate predictor (i.e., missing data were handled by pairwise deletion). We used Fisher’s exact test for binary variables and two-sample equal-variance *t*-tests for the continuous variables (i.e., laboratory measures) differing according to chronic case status; we report two-tail hypothesis test *p* values. The Fisher’s exact test and *t*-tests were implemented using Stata version 16.1 M/P software. Because there are likely to be an inflated Type I error rate when separately considering *p* values from multiple hypothesis tests [48,49], we therefore considered whether the post-hoc test results were Bonferroni-significant accounting for the number of hypothesis tests, assuming these predictors were independent hypothesis tests, by dividing the original significance threshold “*p* < 0.05” by the number of hypothesis tests. We deliberately chose a conservative method for dealing with multiple comparisons because we were aware of a second reason our results in the post-hoc test may be prone to elevated Type I error rates: these post hoc tests were selected conditional an already-fitted model applied to almost the same dataset [50]. These tests assumed independent and identically distributed observations which may be an unrealistic simplifying assumption.

We contrasted the most likely outcome for each participant predicted by the RLLR model against the observed chronic case status of each participant using MedCalc Statistical Software version 19.5.2. We report the model sensitivity, specificity, positive predictive value, negative predictive value, and accuracy [sensitivity * prevalence + specificity * (1-prevalence). We report exact confidence intervals [51] for the sensitivity, specificity, and accuracy, and standard logit confidence intervals [52] for the positive and negative predictive values. We assumed that the prevalence observed in this sample is representative of the prevalence in the general population; similar assumptions about representativeness of our sample also apply to the other analyses reported in this paper.

We then considered the relationships of the variables suggested as predictive of being a chronic by the rigorous logistic lasso to another outcome: the time until cessation of symptoms among chronic ciguatera cases. For this analysis, we implemented a parametric survival analysis using accelerated failure-time regression models [53] assuming proportionate survival times. We conducted sensitivity analyses assuming a lognormal distribution of survival times vs. assuming a Weibull distribution of survival times. The predictors in the survival analysis models were standardized. Results are presented as survival time ratios per one unit change in predictor, with bootstrap percentile 95% confidence intervals [54], with 500 bootstrap replicates. These confidence intervals were selected due to their often-good performance at small sample sizes but ignore possible clustering of participants. These survival analyses were implemented using Stata version 16.1 M/P software.

## Figures and Tables

**Table 1:** Participant’s characteristics.

	All Participants% (n = 49)	Acute CP Cases (n = 26)	Chronic CP Cases (n = 23)
Median age	50	47	52
Sex ratio (M/F)	1.882	1.364	2.833
Ethnicity:			
*Polynesian*	65% (n = 32)	58% (n = 15)	74% (n = 17)
*Mixed-race*	26% (n = 13)	31% (n = 8)	22% (n = 5)
*Non-Polynesian*	8% (n = 4)	11% (n = 3)	4% (n = 1)
Tobacco consumption	43% (n = 21)	23% (n = 6)	65% (n = 15)
Comorbidities:	31% (n = 15)	11% (n = 3)	52% (n = 12)
*Chronic hypertension*	16% (n = 8)	11% (n = 3)	22% (n = 5)
*Type 2 Diabetes*	16% (n = 8)	4% (n = 1)	30% (n = 7)

Italics are used to better distinguish the subpopulations in the different groups (Ethnicity and Comorbidities).

**Table 2:** Characteristics of the full n = 49 participants vs. the n = 38 participants included in the RLLR model.

Variable	All Participants with Data (n = 49)	RLLR Model Sample (n = 38)
Age	50 (45, 60)	49.5 (45, 56)
Male sex	65%	71%
Ate non-herbivorous fish	89% *	89%
Ate head and/or viscera	79% **	82%
Prior ciguatera	57%	63%
Tobacco consumption	43%	45%
Chronic hypertension	16%	16%
Type 2 diabetes	16%	13%
Symptoms incubation time ≥12 h	10%	11%
**Acute Digestive disorders**		
Diarrhea	90%	92%
Vomiting	71%	74%
Abdominal pain	69%	66%
Nausea	53%	50%
**Acute Cardiovascular disorders**		
Hypotension	80%	82%
Bradycardia	55%	58%
Heart rhythm disorder	16%	18%
Tachycardia	4%	3%
**Acute Neurological and Other Disorders**		
Asthenia	88%	87%
Paraesthesia	84%	79%
Itching	71%	66%
Cold allodynia	69%	71%
Dizziness	59%	63%
Migraine	53%	47%
Dysesthesia	51%	45%
Gait disorder	51%	50%
Chill	45%	42%
Muscle disorder	43%	37%
Joint pain	39%	34%
Blurred vision	39%	34%
Hypothermia	35%	37%
Balance disorder	35%	32%
Oral/peri-oral burning sensation	24%	21%
Urogenital pain/itching or discomfort	22%	18%
Dysgeusia	20%	24%
Language disorder	18%	18%
Coordination disorder	14%	11%
Heat allodynia	6%	8%
Dysuria	4%	0%
Depression	2%	0%
**Biological Tests**		
Urea (g/L) [0.13–0.43] ***	0.48 (0.41, 0.56)	0.50 (0.41, 0.57)
Creatinine (mg/L) [6–12]	12 (10, 15)	12.5 (10, 15)
Sodium (mEq/L) [136–145]	140 (138, 141)	140 (138, 141)
Potassium (mEq/L) [3.4–4.4]	4.1 (3.7, 4.4)	4.1 (3.9, 4.4)
Chloride (mEq/L) [98–107]	105 (103, 108)	106 (103, 109)
Leukocytes (g/L) [4.0–10.7]	9.7 (7.4, 11.7)	9.75 (7.7, 11.9)
Neutrophils (g/L) [1.8–7.3]	7.1 (4.4, 8.8)	7.3 (4.8, 8.8)
Eosinophils (g/L) [0.0–0.6]	0.2 (0.1, 0.2)	0.15 (0.1, 0.2)
Basophils (g/L) [0.0–0.2]	0 (0, 0.1)	0 (0, 0.1)
Lymphocytes (g/L) [1.0–3.4]	2 (1.4, 2.6)	1.9 (1.4, 2.6)

* n = 46. ** n = 48. *** normal ranges.

**Table 3:** Frequency of chronic symptoms in the sample of chronic CP cases (n = 23).

Chronic Symptoms	Category	% (n = 23)
Itching	N	65 (n = 15)
Asthenia	N	61 (n = 14)
Paraesthesia	N	56 (n = 13)
Cold allodynia	N	39 (n = 9)
Dysesthesia	N	26 (n = 6)
Muscle disorder	N	26 (n = 6)
Blurred vision	N	26 (n = 6)
Sleep disorder	Np	22 (n = 5)
Diarrhea	D	17 (n = 4)
Joint pain	O	17 (n = 4)
Irritability	Np	17 (n = 4)
Gait disorder	N	17 (n = 4)
Headache/Migraine	N	13 (n = 3)
Hypothermia	N	13 (n = 3)
Chill	N	13 (n = 3)
Peak of hypotension	Cv	13 (n = 3)
Vomiting	D	13 (n = 3)
Urogenital discomfort/pain/burning	O	13 (n = 3)
Abdominal pain	D	9 (n = 2)
Nausea	D	9 (n = 2)
Heart rhythm disorder	Cv	9 (n = 2)
Language disorder	N	9 (n = 2)
Balance disorder	N	9 (n = 2)
Depression	Np	9 (n = 2)
Bradycardia	Cv	4 (n = 1)
Dizziness	N	4 (n = 1)
Coordination disorder	N	4 (n = 1)
Dysgeusia	O	4 (n = 1)
Oral/peri-oral burning sensation	N	4 (n = 1)
Attention disorder	Np	4 (n = 1)

N: neurologic; Np: neuropsychiatric; D: digestive; Cv: cardiovascular; O: other.

**Table 4:** Chronic CP manifestations triggering/worsening factors.

Triggering Factors	% (n = 23)
Food Origin	
Fish	56
Chicken	30
Beef	26
Nuts	22
Pork	17
Soy related products	17
Canned products	17
Dairy products	17
Alcohol	13
Coffee/Tea	9
Chocolate	9
Spices	4
Non-Food Origin	
Cold/hot air contrast	17
Rapid weight loss	17
Physical activity	13
Sun exposure	13

**Table 5:** Significant variables for chronic CP predictors.

Chronic CP Predictors	N	*p* Value (Fisher’s Exact or *t* Test)
Age	49	0.10
Tobacco consumption	49	0.004
Bradycardia	49	0.25
Urea	45	0.03
Neutrophils	45	0.12

**Table 6:** Two-by-two table of the observed chronic case status vs. the predicted chronic case status from the RLLR model.

	Observed Chronic	Observed Non-Chronic
**Predicted Chronic**	n = 24	n = 12
**Predicted Non-Chronic**	n = 2	n = 11

**Table 7:** Parametric survival analyses of time until cessation of symptoms among chronic cases.

Standardized Predictor	n of Participants, n of Events, Person-Months at Risk	Weibull Assumption Time Ratio (Bootstrap 95% CI)	Lognormal Assumption Time Ratio (Bootstrap 95% CI)
Age	23, 16, 535	1.39 (0.65, 3.02)	1.51 (0.55, 2.67)
Tobacco consumption	23, 16, 535	1.19 (0.79, 2.62)	1.38 (0.70, 2.48)
Bradycardia	23, 16, 535	1.31 (0.82, 2.81)	1.60 (0.81, 2.76)
Urea	20, 13, 450	0.71 (0.33, 0.95)	0.72 (0.30, 1.11)
Neutrophils	21, 15, 528	0.94 (0.49, 1.53)	0.88 (0.54, 2.03)
